# The economic costs of malaria in pregnancy: evidence from four sub-Saharan countries

**DOI:** 10.12688/gatesopenres.14375.1

**Published:** 2023-02-15

**Authors:** Laia Cirera, Charfudin Sacoor, Martin Meremikwu, Louise Ranaivo, Manu F. Manun’Ebo, Dachi Arikpo, Osvaldo Matavele, Victor Rafaralahy, Didier Ndombe, Clara Pons Duran, Maximo Ramirez, Francesco Ramponi, Raquel González, Christina Maly, Elaine Roman, Elisa Sicuri, Franco Pagnoni, Clara Menéndez

**Affiliations:** 1 Barcelona Institute for Global Health (ISGlobal), Barcelona, Spain; 2 Manhiça Health Research Center, Manhiça, Mozambique; 3 Cross River Health and Demographic Surveillance System, University of Calabar, Calabar, Nigeria; 4 Malagasy Associates for Numerical Information and Statistical Analysis (MANISA), Antananarivo, Madagascar; 5 Bureau d’Étude et de Gestion de l’Information Statistique (BEGIS), Kinshasa, Democratic Republic of the Congo; 6 Institute of Tropical Diseases Research and Prevention, University of Calabar Teaching Hospital, Calabar, Nigeria; 7 CIBER Epidemiología y Salud Pública (CIBERESP), Barcelona, Spain; 8 Jhpiego, a Johns Hopkins University affiliate, Baltimore, USA; 9 London School of Economics and Political Science, London, UK

**Keywords:** malaria in pregnancy; economic burden malaria; household costs; health system costs

## Abstract

Background

Malaria in pregnancy is a major public health problem in sub-Saharan Africa (SSA), which imposes a significant economic burden. We provide evidence on the costs of malaria care in pregnancy to households and the health system in four high-burden countries in SSA.

Methods

Household and health system economic costs associated with malaria control in pregnancy were estimated in selected areas of the Democratic Republic of Congo (DRC), Madagascar (MDG), Mozambique (MOZ) and Nigeria (NGA). An exit survey was administered to 2,031 pregnant women when leaving the antenatal care (ANC) clinic from October 2020 to June 2021. Women reported the direct and indirect costs associated to malaria prevention and treatment in pregnancy. To estimate health system costs, we interviewed health workers from 133 randomly selected health facilities. Costs were estimated using an ingredients-based approach.

Results

Average household costs of malaria prevention per pregnancy were USD6.33 in DRC, USD10.06 in MDG, USD15.03 in MOZ and USD13.33 in NGA. Household costs of treating an episode of uncomplicated/complicated malaria were USD22.78/USD46 in DRC, USD16.65/USD35.65 in MDG, USD30.54/USD61.25 in MOZ and USD18.92/USD44.71 in NGA, respectively. Average health system costs of malaria prevention per pregnancy were USD10.74 in DRC, USD16.95 in MDG, USD11.17 in MOZ and USD15.64 in NGA. Health system costs associated with treating an episode of uncomplicated/complicated malaria were USD4.69/USD101.41 in DRC, USD3.61/USD63.33 in MDG, USD4.68/USD83.70 in MOZ and USD4.09/USD92.64 in NGA. These estimates resulted in societal costs of malaria prevention and treatment per pregnancy of USD31.72 in DRC, USD29.77 in MDG, USD31.98 in MOZ and USD46.16 in NGA.

Conclusions

Malaria in pregnancy imposes a high economic burden on households and the health system. Findings emphasize the importance of investing in effective strategies that improve access to malaria control and reduce the burden of the infection in pregnancy.

## Key questions

• **What is already known on this topic**?
- It has been argued that households’ costs associated with malaria treatment and prevention are important barriers to healthcare attendance.
- Studies providing detailed evidence on the economic costs of malaria in pregnancy to the households and the health system in SSA are scarce and outdated.
• **What are the new findings?**
- We investigated the economic costs to the households and the health system of malaria control in pregnancy in four endemic countries in SSA, with various contexts and endemicity levels among many other cultural differences and backgrounds.
- Despite a commitment to universal healthcare in study countries, households face a significant financial burden associated to malaria in pregnancy, which is likely to impact the effectiveness of existing malaria control strategies.
• **How this study might affect research, practice or policy**
- Results from this multi-country study will emphasize the importance of investing in strategies to improve access to malaria control tools and reduce the burden of the infection in pregnancy.

## Introduction

Malaria in pregnancy (MiP) is a major contributor to maternal and neonatal mortality and morbidity in sub-Saharan Africa (SSA) ^1^. Recommended strategies for MiP prevention in SSA by the World Health Organization (WHO), include insecticide-treated bed nets (ITNs) and provision of intermittent preventive treatment (IPTp) with sulfadoxine-pyrimethamine (SP) to all women from their second trimester of pregnancy ^2^.

Despite WHO’s recommendations and the strive for universal access to maternal health services, the coverage of these interventions remains low. According to the latest World Malaria Report, the coverage of 3 or more doses of IPTp (IPTp3+) is on average 32% in SSA ^1^.

Several barriers may affect the access and use of malaria control interventions in pregnancy, including difficult access to health facilities, sub-optimal quality of care and low availability of drugs ^3, 4^. Household costs (direct and indirect) associated with malaria control may also constitute a significant barrier ^5– 7^. Results from a meta-analysis described direct medical costs, acquisition of ITNs, and costs of drugs, diagnostic tests and registration fees, as important limitations to accessing malaria control tools in many SSA countries ^3^. Non-medical costs associated with routine antenatal care (ANC) visits, such as food, transport and opportunity costs of patients’ time, pose additional barriers leading to poor ANC attendance and low IPTp uptake in several contexts ^8^.

Nevertheless, available estimates on costs of treating and preventing MiP are outdated and only available in some specific contexts, compared to other malaria interventions ^9, 10^. Representative and detailed up-to-date costing estimates of malaria in pregnancy in SSA, are essential to conduct further economic evaluations of malaria interventions, inform policy decisions and improve allocation of resources in the region. 

This study aims at providing accurate estimates on the economic costs of malaria treatment and control in pregnancy to the households and the health system in high endemic areas. The study focuses on the Democratic Republic of Congo (DRC), Madagascar (MDG), Mozambique (MOZ) and Nigeria (NGA), countries which accounted for nearly half of worldwide malaria deaths in 2020 ^1^.

## Methods

### Ethical considerations

Written informed consent was sought from all individuals who participated in the ANC exit survey and the questionnaire to the health workers, before conducting any study procedures. All study protocols and informed consents were approved by the Ethics Committee of the Hospital Clinic in Barcelona, the WHO ethics review board [(ERC.0003384 – 02/10/2020; (CCI/051/AGO/2020 – 20/08/2020)] 

### Study setting

This costing study was part of a multi-country project to assess the effectiveness and cost-effectiveness of community-based delivery of intermittent preventive treatment (C-IPTp) of malaria during pregnancy ^11^. The study was conducted in 12 rural districts in four SSA countries: DRC, MDG, MOZ and NGA. The selection of these countries was based on criteria that included the existence of an operative system of community health workers (CHW), having IPTp policies in place, commitment from the Ministry of Health (MoH) and high malaria endemicity. The study areas were heterogeneous in terms of the demographic and health profiles, but all them were endemic of malaria, with the disease being among the leading causes of maternal and child mortality ^11^.

### Study design and participants

The economic costs of malaria in pregnancy include the cost to the health system that provides prevention and treatment services, and the cost to households who access these services.

To collect the household costs of malaria in pregnancy, we administered an exit survey questionnaire to pregnant women when leaving a routine ANC visit from October 2020 to June 2021. Sample size for the minimum number of pregnant women to be interviewed was calculated as follows: *n ≥ Z _α_^2 (p(1-p))/e^2*, where *Z* is the critical value of the normal distribution at level *α=0.05* (95% confidence level), *e* is the margin of error ( *e*=0.05) and *p* is the households cost variability ^12^. Minimum sample size, assuming a 10% of dropout rate, resulted in 426 pregnant women interviewed per country.

To estimate the health system costs associated to control of MiP, interviews were administered to health workers from a random sample of 30% selected health facilities of existent facilities within the district. This resulted in a total of 133 health workers from 133 different health facilities being approached and interviewed, with any participant declining or withdrawing later on from the interview (see Table S1 ^13^).

### Household costs

All women leaving an ANC visit were approached for an interview. For those women that met the inclusion criteria, (i.e., being pregnant, living in the study area and leaving an ANC consultation), we sought written consent to participate. Participants were asked about the direct and indirect costs associated to attending a routine ANC visit, where malaria prevention services are provided. Direct costs (out-of-pocket expenses) were broken into medical (e.g., drugs and tests, registration fees), and non-medical costs (transportation and food while at health facility). Indirect costs reflected the value of the time lost due to attending an ANC visit. The average household cost of malaria prevention per pregnancy was calculated by multiplying the cost of providing malaria prevention services through an ANC visit by the average number of IPTp doses received ^14^.

Women who reported having experienced an episode of malaria in their current pregnancy (n=434) (Table S1 ^13^), were asked about the direct (medical and non-medical) and indirect (value of time lost because of illness) costs associated to malaria treatment they had incurred. Uncomplicated malaria was defined as a confirmed malaria episode diagnosed at the outpatient clinic and not requiring hospital admission, while complicated malaria was defined as an episode of malaria requiring hospital admission. For inpatients cases, women were enquired about the costs incurred during hospitalization, length of stay and the presence of a caregiver while hospitalized and after discharge at home. The monetary value of women’s time lost was estimated by taking into consideration the average wage by activity sector and country ( https://meusalario.org/mocambique/salario/sector-publico-mocambique/salarios-do-sector-de-saude) ^15– 21^. For unemployed participants, studying or working in the informal sector, the minimum wage per country was considered. For women who had a caregiver while being admitted at the hospital or at home, the caregiver’s value of time lost was also included.

### Health system costs

The health system costs associated to preventing malaria were retrieved and consist of the provision of IPTp and ITNs at the ANC visits. These costs included the costs of IPTp treatment with SP, the distribution of ITNs at the first ANC visit, personnel time and facilities running costs. Health personnel costs and health facility running costs were allocated to malaria prevention services based on an assessment of the proportion of time devoted to these services during an ANC visit. Reference prices for drugs and mosquito nets were taken from WHO and the Global Fund procurement prices. The average prevention costs per pregnancy were approximated by multiplying the costs of an ANC visit by the average number of IPTp doses received per women in intervention areas ^14^.

In addition, the health system costs of treating an episode of MiP, both for uncomplicated and complicated malaria, were estimated. The average cost of an uncomplicated case was defined as the costs of managing a malaria case in pregnancy as an outpatient, while the average costs of a complicated malaria episode were approximated by the average cost of a hospital admission case.

Recurrent (personnel salaries and time, medical supplies, etc.) and capital costs (utilities and running costs) associated with malaria treatment in pregnancy were estimated based on the average time and clinical staff involved in the management of MiP. WHO and the Global Fund procurement prices were used as reference prices for drugs, tests and vaccines ^22, 23^. To estimate health facilities’ running costs, overall monthly expenses were allocated to a malaria case or an ANC visit, based on the proportion of malaria episodes or ANC visits of the total of outpatient visits. For health facilities with inpatient services (n=30), the cost per inpatient bed day was obtained from the WHO estimates ^24^. Total admission costs were calculated by multiplying the cost per inpatient bed day by the average number of admission days reported.

### Societal costs

The societal cost of malaria care per pregnancy was estimated by including the costs of malaria prevention and treatment to the health system and households. The average treatment costs per pregnancy, both for the health system and households, were calculated by multiplying the treatment costs per malaria case (complicated and non-complicated) by the incidence of malaria (complicated and non-complicated) in pregnancy. We approximated the incidence of MiP by using self-reported data from pregnant women in intervention areas (Table S1 ^13^).

### Data management and analysis

Data was collected through standardized questionnaires using REDCap and data was directly entered after asking questions verbally to participants. Alternative applications to REDCap which are available for free include Qualtrics, among others *.* . Stata 17 and Microsoft Excel 2019 were used to perform the costing calculations. Spearman’s rank correlation coefficient was used to assess the association between households’ time lost and overall costs associated to malaria control and treatment in pregnancy.

## Results

### Household costs

Out of the 2,617 pregnant women approached, 2,031 met the inclusion criteria and were interviewed (Table S1 ^13^). The mean participant’s age was 24 years (SD 6.4), with more than 80% of them being married or in union (Table S2 ^13^). Most of the participants worked as subsistence farmers, except in NGA where almost 45% of respondents worked as small-scale traders or self-employed workers. The level of education varied by country, with DRC having the highest share of respondents with secondary and higher studies accomplished (67.7%), and Mozambique the lowest (9.6%). The percentage of women reporting having experienced an episode of malaria in their current pregnancy ranged between 8.8% in Madagascar and 42.7% in Nigeria.

Table 1 shows household costs associated to malaria prevention in pregnancy. The average household costs of attending an ANC visit, where malaria preventive measures in pregnancy are provided, such as ITNs or IPTp, were multiplied by the average IPTp doses received, resulting in average household prevention costs per pregnancy of USD6.33 in DRC, USD10.06 in MDG, USD15.03 in MOZ and USD13.33 in NGA. The indirect cost – time lost due to attending an ANC visit, with an average time loss of 3.5 hours/ANC visit – was the main contributor.

**Table 1.** Household costs of malaria prevention in pregnancy (USD 2018).

Variable	Household costs of malaria prevention in pregnancy (USD 2018)							
	DRC (n=450)		MDG (n=556)		MOZ (n=543)		NGA (n=482)	
	Mean	SD	Mean	SD	Mean	SD	Mean	SD
**Direct costs (out of pocket)**	**0.41**	**(0.87)**	**0.41**	**(0.61)**	**0.63**	**(1.99)**	**1.10**	**(1.67)**
Direct non-medical costs ^1^	0.07	(0.47)	0.10	(0.46)	0.58	(1.98)	0.68	(1.23)
Direct medical costs ^2^	0.34	(0.73)	0.32	(0.44)	0.05	(0.22)	0.42	(1.05)
**Indirect cost** **(Value of time lost)** ^3^	**1.94**	**(1.39)**	**1.78**	**(1.18)**	**4.76**	**(2.68)**	**2.34**	**(1.97)**
Transport time (go and back)	108	minutes	158	minutes	197	minutes	63	minutes
Time at HF (waiting + consultation)	101	minutes	99	minutes	121	minutes	105	minutes
Value of 1 minute lost	0.009		0.007		0.015		0.014	
**Household costs malaria prevention per ANC visit**	**2.34**	**(1.73)**	**2.19**	**(1.25)**	**5.38**	**(2.9)**	**3.44**	**(1.38)**
**Average IPTp doses**	**2.70**		**4.58**		**2.79**		**3.87**	
**Household costs malaria prevention per pregnancy**	**6.33**	**(4.68)**	**10.06**	**(5.90)**	**15.03**	**(8.67)**	**13.33**	**(11.62)**

^1^ Includes travel costs to/from the health facility and non-medical costs at health facility (i.e., food and water, registration fees). In DRC, MDG, MOZ and NGA, 96%, 94%, 96% and 79% of women reported having walked to the HF, respectively ^2^ Includes medical costs at the health facility. In DRC, MDG, MOZ and NGA, 22%, 54%, 12% and 33% of women reported having spent some money at the HF, respectively ^3^ It includes the value of the time lost due to attending the ANC visit to receive malaria prevention services (transport+waiting time+consultation).

Overall, out of the 2,031 interviewed pregnant women, 73 (4%) experienced an episode of complicated malaria in their current pregnancy and 361 (18%) referred to having had an episode of uncomplicated malaria (Table S1 ^13^). Household average cost associated with an episode of uncomplicated malaria was USD22.78 in DRC, USD16.65 in MDG, USD30.54 in MOZ and USD18.92 in NGA ( Table 2). Regarding complicated malaria, household costs were USD35.65 in MDG, USD44.71 in NGA, USD46 in DRC and USD61.25 in MOZ ( Table 3).

**Table 2.** Household costs associated to an episode of uncomplicated malaria (USD 2018).

Variable	Household costs associated to an episode of uncomplicated malaria (USD 2018)							
	DRC (n=63)		MDG (n=42)		MOZ (n=81)		NGA (n=75)	
	Mean	SD	Mean	SD	Mean	SD	Mean	SD
**Direct costs of care-seeking**	**6.09**	**(5.09)**	**2.95**	**(2.89)**	**0.94**	**(1.93)**	**4.41**	**(4.44)**
Direct non-medical costs ^1^	1.13	(3.86)	0.86	(1.57)	0.78	(1.73)	0.64	(1.52)
Direct medical costs ^2^	4.96	(4.48)	2.09	(2.41)	0.16	(0.60)	3.78	(4.02)
**Indirect cost (Value of time lost) ^3^**	**16.69**	**(16.2)**	**13.70**	**(14.01)**	**29.60**	**(17.56)**	**14.51**	**(18.42)**
	*Minutes*		*Minutes*		*Minutes*		*Minutes*	
Transport time (go and back)	101		136		169		58	
Time at HF (waiting + consultation)	121		57		100		92	
Value of 1 minute lost	0.010		0.007		0.015		0.015	
**Household costs per episode of** **uncomplicated MiP**	**22.78**	**(18.99)**	**16.65**	**(14.71)**	**30.54**	**(17.87)**	**18.92**	**(19.61)**

DRC, Democratic Republic of Congo; MDG, Madagascar; MOZ, Mozambique; NGA, Nigeria; SD, standard deviation; HF, health facility; MIP, malaria in pregnancy. ^1^ Includes travel costs to/from the health facility and non-medical costs at health facility (e.g., food and water) ^2^ Includes medical costs at the health facility. ^3^ It includes the value of the time lost due to having an episode of malaria (transport, waiting time, diagnosis and consultation), as well as the value of the income foregone due to being unable to perform the normal economic activity. It also includes the loss of income for the caregiver. See table S3 ^13^) for details.

**Table 3.** Household costs associated to an episode of complicated malaria (USD 2018).

Variable	Household costs associated to an episode of complicated malaria (USD 2018)							
	DRC (n=33)		MDG (n=7)		MOZ (n=2)		NGA (n=31)	
	Mean	SD	Mean	SD	Mean	SD	Mean	SD
**Direct costs**	**13.522**	**(5.09)**	**8.08**	**(2.89)**	**4.31**	**(1.93)**	**16.4**	**(4.44)**
Direct non-medical costs ^1^	5.66	(3.86)	2.76	(1.57)	4.27	(1.73)	2.37	(1.52)
Direct medical costs ^2^	7.86	(4.48)	5.32	(2.41)	0.04	(0.60)	14.04	(4.02)
**Indirect costs (Value of time lost) ^3^**	**32.48**	**(18.99)**	**27.57**	**(14.71)**	**56.94**	**(8.33)**	**28.30**	**(23.28)**
	*Minutes/days*		*Minutes/days*		*Minutes/days*		*Minutes/days*	
Transport time (go and back)	118	minutes	240	minutes	285	minutes	88	minutes
Days hospitalized	4	days	2	days	4	days	2	days
Value of 1 minute lost	0.009		0.009		0.015		0.014	
**Household costs per episode of** **complicated MiP**	**46.00**	**(23.44)**	**35.65**	**(11.18)**	**61.25**	**(6.89)**	**44.71**	**(33.79)**

DRC, Democratic Republic of Congo; MDG, Madagascar; MOZ, Mozambique; NGA, Nigeria; SD, standard deviation; MIP, malaria in pregnancy. ^1^ Includes travel costs to/from the health facility and non-medical costs at health facility (e.g., food and water) ^2^ Includes medical costs at the health facility and treatment/drug costs after the health facility visit. ^3^ It includes the value of the time lost due to having an episode of complicated malaria and the loss of income for the caregiver. See table S3 ^13^ for details.

**Figure 1.**
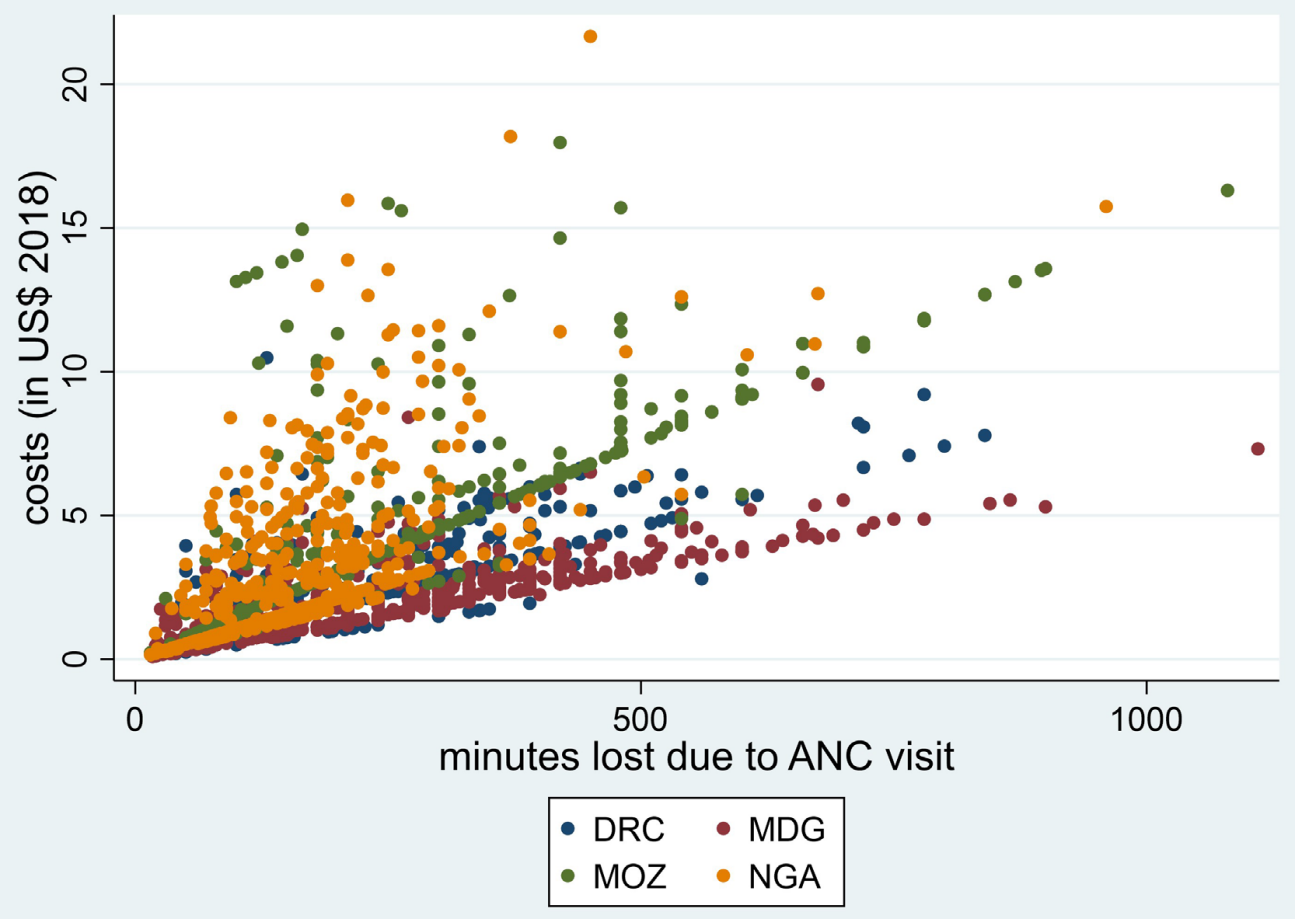
Association between minutes lost due to receiving malaria preventive services at the ANC and household costs (in USD). ANC, antenatal care; DRC, Democratic Republic of Congo; MDG, Madagascar; MOZ, Mozambique; NGA, Nigeria.

**Figure 2.**
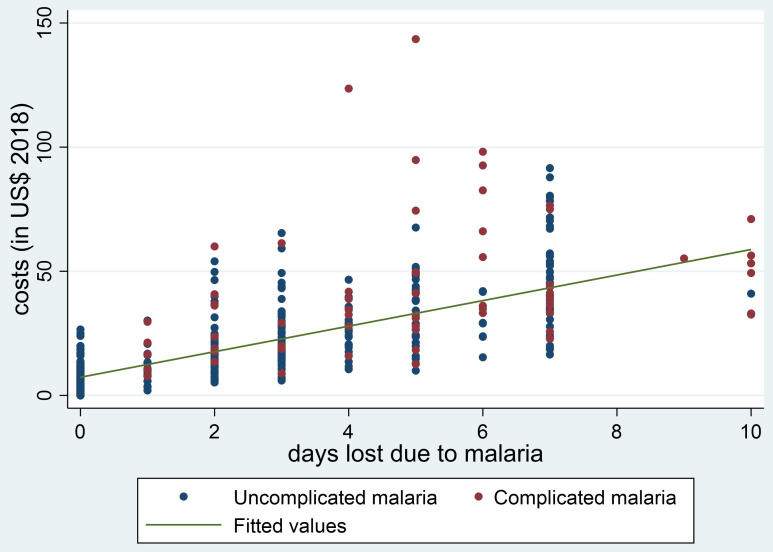
Association between days lost due to being ill with malaria and household costs (in USD). DRC, Democratic Republic of Congo; MDG, Madagascar; MOZ, Mozambique; NGA, Nigeria.

The indirect cost of treatment was the highest cost, both for complicated and uncomplicated malaria episodes ( Table 2, Table 3 and Table S3 ^13^). In Figure 1 and Figure 2, the plots of the total minutes lost due to an ANC visit ( Figure 1) and the days lost due to an episode of malaria ( Figure 2) against the overall households’ costs are shown. In both plots there is a strong association between the time lost and the overall households’ costs, with a Spearman’s rank correlation coefficient of 0.73.

Out-of-pocket (OOP) expenses, particularly the direct medical expenses associated to treatment and prevention of malaria in pregnancy, were found to be present across study countries ( Table 2 and Table 3). More specifically, 22% (14 out of 63) of the women interviewed in DRC, 54% (23 out of 42) in MDG, 12% (10 out of 81) in MOZ and 33% (58 out of 175) in NGA, reported direct medical expenses when having an outpatient visit related to an uncomplicated malaria episode (e.g., tests, drugs and registration fees). For the admission cases, all women reported having incurred in direct medical expenses in all study sites.

### Health system costs

The health centres included in the survey were all part of the public health system but significantly differed in terms of population catchment area, human resources and equipment (Table S4 ^13^). Among selected health facilities, 23% (30 out of 133) had the resources to treat an episode of complicated malaria in pregnancy (i.e., inpatient services), while in 77% of them (103 out of 133) patients with complicated malaria had to be transferred to the district hospital or other referral centres.

Table 4 presents the health system costs of preventing MiP through the ANC visits. The average health system costs per pregnancy were USD10.74 in DRC, USD16.95 in MDG, USD11.17 in MOZ and USD15.64 in NGA. Drugs (IPTp treatment with SP) constituted the largest share of cost, followed by insecticide treated nets (ITNs). 

**Table 4.** Health systems costs of preventing MiP (USD 2018).

Variable	Health systems costs of preventing MiP (USD 2018)							
	DRC (n=26)		MDG (n=35)		MOZ (n=20)		NGA (n=52)	
	Mean	%	Mean	%	Mean	%	Mean	%
Fansidar/SP	3.49	100%	3.49	100%	3.49	100%	3.49	100%
Mosquito nets ^1^	0.65	100%	0.65	100%	0.65	100%	0.65	100%
Health personnel ^2^	0.64	30%	0.18	30%	0.43	30%	0.92	30%
Utilities and running costs ^3^	0.18	30%	0.05	30%	0.50	30%	0.36	30%
**Health system cost per ANC visit**	**4.39**	**(0.36)**	**4.21**	**(0.064)**	**4.42**	**(0.23)**	**4.53**	**(0.47)**
**Average IPTp doses**	**2.70**		**4.58**		**2.79**		**3.87**	
**Health system cost per pregnancy**	**10.74**		**16.95**		**11.17**		**15.64**	

^1^ Mosquito nets were reported to be distributed at the first ANC visit in all study sites. ^2^ Based on average time of an ANC visit (34 min in DRC, 15 min in MDG, 24 min in MOZ and 42 min in NGA). 30% of health personnel costs allocated to malaria prevention services based on assessment of the proportion of time devoted to these services. ^3^ Monthly average costs of running the HF in each country were divided by the average monthly number of ANC visits. In addition, 30% of ANC overall running costs allocated to malaria prevention services based on assessment of the proportion of time devoted to these services.

Provider costs for the treatment of an uncomplicated malaria episode in pregnancy were USD4.69 in DRC, USD3.61 in MDG, USD4.68 in MOZ and USD4.09 in NGA ( Table 5). Case management of uncomplicated malaria was similar across study countries, with rapid diagnostic test (RDTs) being used as the main diagnostic tool, and artemether-lumefantrine as the main first-line drug for treatment of uncomplicated malaria (Table S5 ^13^).

**Table 5.** Health system costs associated with management of an episode of uncomplicated malaria in pregnancy (USD 2018).

Variable	Health systems costs associated to managing an episode of non- complicated MiP (USD 2018)							
	DRC (n=26)		MDG (n=35)		MOZ (n=20)		NGA (n=52)	
	Mean	(SD)	Mean	(SD)	Mean	(SD)	Mean	(SD)
Drugs (including malaria treatment)	2.80	(2.3)	2.17	(2.3)	2.02	(1.32)	1.71	(1.4)
Lab. and diagnostic tests ^1^	0.94	(0.4)	0.94	(0.3)	0.94	(0.42)	0.94	(1.6)
Health personnel ^2^	0.68	(0.34)	0.36	(0.2)	0.74	(0.2)	1.08	(0.4)
Utilities and running costs ^3^	0.27	(0.33)	0.13	(0.1)	0.99	(0.4)	0.37	(0.55)
**Total costs episode non-complicated** **MiP**	**4.69**	**(0.30)**	**3.61**	**(0.13)**	**4.68**	**(0.25)**	**4.09**	**(0.53)**

^1^ 99% of HFs use RDT as main diagnostic test. See table S5 for details. ^2^ Based on the average time of managing an episode of non-complicated malaria (36 min in DRC, 30 min in MDG, 46 min in MOZ and 49 min in NGA) ^3^ Monthly average costs of running the HF in each country were divided by the average monthly episodes of non-complicated malaria

Regarding complicated malaria, the treatment costs per episode were USD101.41 in DRC, USD63.33 in MDG, USD83.70 in MOZ and USD92.64 in NGA ( Table 6). Differences in costs across countries were due to differences in hospital admission costs and number of admission days, with total admission costs per malaria case of USD88.16 in DRC, USD47.49 in MDG, USD64.20 in MOZ and USD77.33 in NGA ( Table 6). Further details on the management of a complicated malaria episodes in pregnancy are presented in supplementary Table S6 ^13^).

**Table 6.** Health system costs associated with management of an episode of complicated malaria in pregnancy (USD 2018).

Variable	Health systems costs associated to managing an episode of non- complicated MiP (USD 2018)							
	DRC (n=4)		MDG (n=15)		MOZ (n=5)		NGA (n=6)	
	Mean	(SD)	Mean	(SD)	Mean	(SD)	Mean	(SD)
Malaria treatment	9.16	(7.75)	11.49	(7.74)	15.08	(0.73)	10.57	(6.60)
Other drugs	2.21	(0.00)	2.02	(1.46)	2.54	(1.00)	2.86	(1.40)
Lab. and diagnostic tests ^1^	1.88	(0.00)	1.88	(0.00)	1.88	(0.00)	1.88	(0.00)
Hospital admission costs ^2^	88.16	(48.81)	47.94	(18.45)	64.20	(0.2)	77.33	(18.24)
**Total costs episode non-** **complicated MiP**	**101.41**	**(41.7)**	**63.33**	**(19.78)**	**83.70**	**(1.72)**	**92.64**	**(24.15)**

^1^ 99% of HFs use RDT as main diagnostic test. See table S6 ^13^ for details.

^2^ Cost per inpatient bed day for primary hospitals obtained from WHO-CHOICE estimates (2010). Values updated to USD 2018. Estimates based on 80% occupancy rate, excluding drugs and diagnostics. It includes personnel costs while admitted at the hospital. We took the reference parameter for eastern and western Africa, respectively.

### Societal costs

As presented in Table 7, mean societal costs of malaria prevention and treatment per pregnancy were USD31.72 in DRC, USD29.77 in MDG, USD31.98 in MOZ and USD46.16 in NGA. If we assume that the proportion of pregnant women represents 4% of the total population, this would translate, for a targeted area with 100,000 inhabitants, into a yearly economic burden of malaria in pregnancy of USD126,874 in DRC, USD119,083 in MDG, USD127,901 in MOZ and USD184,620 in NGA.

**Table 7.** Societal costs of malaria prevention and treatment in pregnancy (USD 2018).

Variable	Societal costs of malaria prevention and treatment in pregnancy (USD 2018)							
	DRC		MDG (n=556)		MOZ (n=543)		NGA (n=482)	
	Mean	SD	Mean	SD	Mean	SD	Mean	SD
**Household costs per pregnancy**	**12.89**	**(4.68)**	**11.76**	**(5.90)**	**19.80**	**(8.67)**	**23.07**	**(11.62)**
Average prevention costs	6.33		10.06		15.03		13.33	
Average treatment costs non-complicated malaria ^1^	3.19		1.25		4.55		6.87	
Average treatment costs complicated malaria ^2^	3.37		0.45		0.23		2.87	
**Health system costs per pregnancy**	**18.83**	**(0.30)**	**18.01**	**(0.09)**	**12.18**	**(0.20)**	**23.09**	**(0.65)**
Average prevention costs	10.74		16.94		11.17		15.64	
Average treatment costs non-complicated malaria ^1^	0.66		0.27		0.70		1.49	
Average treatment costs complicated malaria ^2^	7.43		0.80		0.31		5.96	
**Societal costs of malaria prevention and treatment per pregnancy**	**31.72**	**(4.60)**	**29.77**	**(5.92)**	**31.98**	**(8.66)**	**46.16**	**(11.70)**

^1^ The incidence of self-reported non-complicated malaria during pregnancy was 14%, 7.55%, 14.92% and 36.31% in DRC, MDG, MOZ and NGA, respectively (table S1 ^13^) ^2^ The incidence of self-reported complicated malaria during pregnancy was 7.33%, 1.26%, 0.37% and 6.43% in DRC, MDG, MOZ and NGA, respectively (table S1 ^13^)

## Discussion

In this study we have estimated costs associated with the treatment and prevention of malaria in pregnancy in four endemic countries in SSA from the perspective of the health provider and the households. The results showed that despite the international call to universal healthcare ^25^, in endemic SSA countries pregnant women and their families experienced a significant economic burden associated with attending routine ANC clinic visits to receive malaria prevention services and treatment for malaria. These expenses relate to the opportunity costs and the OOP costs, including direct medical and non-medical costs. 

The main driver of household costs was the opportunity costs in terms of the value of the time lost due to care seeking or being ill with malaria. Across countries, it was observed that, including transport, waiting time and time at the consultation, women spent an average of 4 hours attending a routine ANC visit and 3.5 hours when seeking care for a malaria outpatient episode. These figures reflect the households’ barriers in accessing health facilities, as well as the scarcity of resources at the health facilities, resulting in high user-to-staff ratios and long waiting times (Tables S3 and S4 ^13^). Similar results have been reported in comparable contexts ^26^.

These costs represent a high economic burden on households and may constitute a catastrophic cost, especially for the most vulnerable families. Considering the participants’ average monthly wage ‒USD90 in DRC, USD70 in MDG, USD143 in MOZ and USD135 in NGA ‒ the average household costs associated with preventing and treating malaria per pregnancy represent between 22% and 43% of their monthly income.

The findings of this study are in alignment with results from an anthropological study conducted in the same intervention areas, whereby households’ OOP costs were identified as significant barriers to access facility-based care and treatment ^27^. OOP costs were particularly high in NGA, where OOP payments by users are a main mechanism for funding costs of treatments, tests and drugs provided at the health facility ^28^.

When compared to estimates from the literature, the household costs in this study are higher for the following reasons ^5, 6, 29^. First, our estimates assess the value of the time lost (opportunity cost) by considering the wage of each woman in her specific sector of employment, while in the other studies the minimum average wage or the GDP per capita in each country was used. In addition, in this study the time lost for the women was valued, as well as the caregiver’s value of time, when present. Finally, the estimates used in other studies for household costs referred to average malaria treatment costs across the whole population, while in this study we used the treatment costs specific to malaria in pregnancy. Other literature examining treatment costs associated to MiP, point out that treatment costs are significantly higher compared to the average costs for the whole population ^30, 31^.

Health provider costs associated to treatment of malaria in pregnancy were also high. There were no major differences across study countries regarding malaria case management. Drugs constituted the largest share of cost, driven by the administration of IPTp with SP at routine ANC visits. 

Variations in costing estimates for complicated malaria were driven by patient admission costs, hospital daily costs and average length of stay. The average health system costs associated to malaria treatment in pregnancy were higher than the median value found in a meta-analysis, although within the range of published values – USD2.36 to USD23.65 for uncomplicated malaria and USD15.64 to USD137.87 for complicated malaria ^32^. These variances can be explained by differences in health system characteristics in each country, contextual factors and by co-payment mechanisms between patients and providers. 

In this study, data were gathered through a cross-sectional survey conducted among pregnant women when leaving an ANC visit. The costs collected captured the cost of preventive services provided at the ANC visits or outpatient visits but did not capture the complete health care pathway that the women may have received, especially if they suffered from repeated episodes of malaria or had adverse effects from MiP. Therefore, household costs estimated in this study should be considered as a lower bond of the true economic burden incurred by the pregnant women.

## Conclusion

Results from this study demonstrate the significant economic burden that malaria infection imposes on both the household and the health system in endemic countries of SSA. Updated cost estimates from endemic areas are essential to inform economic evaluations for malaria control in pregnancy. Moreover, findings underline the need to explore alternative strategies to overcome the economic burden faced by pregnant women, such as community-based delivery approaches, and the importance of improving access to malaria care in pregnancy.

## Data Availability

figshare: Underlying_data. https://doi.org/10.6084/m9.figshare.21997595.v1 ^33^ This project contains the following underlying data: Data file 1. “Exit_survey_database.csv” Data file 2. “Health_workers_database.csv” Data file 3. “Exit_survey_data_key.xls” Data file 4. “Health_workers_data_key.xls” Data file 5. “Households_costs_analysis.txt” Data file 6. “Health_system_costs_analysis.txt” figshare: Data Questionnaires and supplementary information https://doi.org/10.6084/m9.figshare.21997529.v1 ^13^ This project contains the following extended data: Data file 1. “Exit_survey_questionnaire.doc” Data file 2. “Health_workers_questionnaire.doc” Data file 3. “Supplementary information.doc” Data are available under the terms of the Creative Commons Zero "No rights reserved" data waiver (CC0 1.0 Public domain dedication).
