# Supplementary material for: The economic costs of malaria in pregnancy: evidence from four sub-Saharan countries
Source: Gates Open Res. 2023 May 5;7:47. Originally published 2023 Feb 15. [Version 2] doi: 10.12688/gatesopenres.14375.2 (PMC10205974; doi:10.12688/gatesopenres.14375.2)
Supplement: Peer review history

# Peer review: Reviewer response for version 2

**DOI:** 10.21956/gatesopenres.15992.r33152

**Published:** 2023-05-23

**Authors:** Ann Levin

**Affiliations:** 1 Levin and Morgan LLC, Bethesda, MD, USA

The authors have addressed my comments adequately.

Is the work clearly and accurately presented and does it cite the current literature?

Yes

If applicable, is the statistical analysis and its interpretation appropriate?

Partly

Are all the source data underlying the results available to ensure full reproducibility?

Yes

Is the study design appropriate and is the work technically sound?

Yes

Are the conclusions drawn adequately supported by the results?

Yes

Are sufficient details of methods and analysis provided to allow replication by others?

Yes

Reviewer Expertise:

Health economics

I confirm that I have read this submission and believe that I have an appropriate level of expertise to confirm that it is of an acceptable scientific standard.

# Peer review: Reviewer response for version 1

**DOI:** 10.21956/gatesopenres.15687.r32937

**Published:** 2023-04-03

**Authors:** Ann Levin

**Affiliations:** 1 Levin and Morgan LLC, Bethesda, MD, USA

The paper is about the economic costs of malaria in pregnancy. The paper is well-written. The following are some general comments:

1. The authors say that they are estimating economic costs, but don't define how economic costs differ from financial ones. It would be helpful to define economic costs in the paper. 
2. It is unclear how the districts and facilities were selected for the study in each country. What sampling was done in the selection of these? The authors should explain this so that the reader can assess whether these are representative in their countries.
3. No sensitivity analysis was conducted to account for uncertainty in the variable values. It is likely that there was some uncertainty in variable values or assumptions. Why wasn't this done?

Some specific comments are the following:

1. Pg. 4, second paragraph, do you mean to say that one health worker each from 133 facilities?
2. Pg. 4, fourth paragraph, "Women were enquired about the costs incurred...": 'enquired' could be changed to 'asked'.

Is the work clearly and accurately presented and does it cite the current literature?

Yes

If applicable, is the statistical analysis and its interpretation appropriate?

Partly

Are all the source data underlying the results available to ensure full reproducibility?

Yes

Is the study design appropriate and is the work technically sound?

Yes

Are the conclusions drawn adequately supported by the results?

Yes

Are sufficient details of methods and analysis provided to allow replication by others?

Yes

Reviewer Expertise:

Health economics

I confirm that I have read this submission and believe that I have an appropriate level of expertise to confirm that it is of an acceptable scientific standard, however I have significant reservations, as outlined above.

# Response

**Published:** 2023-04-20

**Authors:** Laia Cirera

**Affiliations:** Barcelona Institute for Global Health, Spain

Thank you for the important comments on this study. Please, see below the answers and the correspondent changes in the manuscript based on your comments:

 The authors say that they are estimating economic costs, but don't define how economic costs differ from financial ones. It would be helpful to define economic costs in the paper. 

- **Response:** Thanks for this relevant comment. Economic costs include both the financial costs (i.e. the direct expenditures on goods and services purchased, such as drugs, tests, transport or food) as well as the indirect cost, that is, the value of the time lost (opportunity cost). In the context of this study, we assumed that the opportunity cost is the women’s value of the productivity lost due to going to the health facility. To better clarify this:
  - In line 12 we changed ‘financial burden’ for ‘economic burden’.
  - The methods section (lines 90-91) now includes the following sentence: “While direct costs reflect the financial costs, the economic costs include both direct and direct costs”    

It is unclear how the districts and facilities were selected for the study in each country. What sampling was done in the selection of these? The authors should explain this so that the reader can assess whether these are representative in their countries.

- **Response:** Thanks for the comment. As specified in line 55, the study is part of a multi-country project to assess the effectiveness and cost-effectiveness of C-IPTp. As such, project countries and districts were selected by the project implementers in collaboration with governments. Selection was based on countries’ geographical diversity across WHO African region, a high burden of malaria, and the commitment of the government to implement C-IPTp, among others. Details on study areas are provided in another paper (Gonzalez et. al – Reference 11), which we have now also referenced in the Study setting (line 63).  In addition, the criteria to conduct the interviews to 30% of existent health facilities was based on the available budget, as this were the estimated maximum number of health facilities that could be reached. The 133 health facilities that were finally approached, were randomly selected within the list of existent health facilities in each district (lines 77-81).

No sensitivity analysis was conducted to account for uncertainty in the variable values. It is likely that there was some uncertainty in variable values or assumptions. Why wasn't this done?

- **Response:** We see the reviewer’s point and we agree that the information reported might be subject to uncertainty. As specified in line 55, this study is part of a a multi-country project to assess the effectiveness and cost-effectiveness of community-based delivery of intermittent preventive treatment (C-IPTp) of malaria during pregnancy. In the cost-effectiveness analysis of the intervention, which is part of a separate manuscript that will follow the publication of this manuscript, we provide accurate sensitivity analysis (one way and probabilistic sensitivity analysis), where key parameters are subject to uncertainty. Bearing this in mind, in this article the focus was rather on providing a disaggregated and accurate description of the economic costs associated to malaria in pregnancy, including the mean and also the standard deviation. These estimates conform the basis for conducting the main cost-effectiveness and sensitivity analysis.  

.Pg. 4, second paragraph, do you mean to say that one health worker each from 133 facilities?-

- **Response:** That’s right. We have edited lines 78 and 79 accordingly in order to clarify this.

Pg 4. fourth paragraph, "Women were enquired about the costs incurred...": 'enquired' could be changed to 'asked'

- **Response:** Thanks, this has been changed as suggested (line 100).

# Peer review: Reviewer response for version 1

**DOI:** 10.21956/gatesopenres.15687.r32939

**Published:** 2023-03-27

**Authors:** Laetitia Duval

**Affiliations:** 1 Centre d’Economie de La Sorbonne, University Paris 1 Panthéon-Sorbonne, Paris, France

The manuscript provides evidence on the costs of malaria care in pregnancy to households and the health system in four countries: Democratic Republic of Congo, Madagascar, Mozambique and Nigeria. Results from this multi-country study emphasize the importance of investing in strategies to improve access to malaria control and reduce the burden of malaria in pregnancy.

 My comments are minor and only need to be clarified.

 I suggest authors to explore more deeply the contexts of these four high-burden countries in SSA to better interpret the results (e.g. seasonality of transmission, major differences across study countries in health system characteristics, contextual factors, co-payment mechanisms between patients and providers, etc.). The Discussion needs to further explain these differences, which represent a rare opportunity to cost malaria care in pregnancy in low-income countries that have not received much attention in the literature. Despite these differences, is there a key message to policy makers emanating from these four settings?

 The exit survey questionnaire to pregnant women has been administered when leaving a routine ANC visit from October 2020 to June 2021. The COVID-19 pandemic could have major adverse effects on the provision of health services for other major infectious diseases, such as malaria. Any assumptions about how the programmes for malaria prevention and treatment have been affected during this period of time? Care seeking reduced compared with pre-pandemic levels, prevention services partially suspended (LLIN mass campaigns halted, mass drug distribution or seasonal malaria chemoprevention interrupted, etc.)?

 Please provide the exclusion criteria of participants: was there an age, co-morbidity limit?

 The costs data have been obtained from a cross-sectional survey, a limitation of the study that has been underlined by the authors. Please explain briefly what data ideally would allow scholars to estimate the economic burden of malaria over the course of the entire malaria season and/or individual pregnancies to better reflect the impact of multiple episodes?

Is the work clearly and accurately presented and does it cite the current literature?

Yes

If applicable, is the statistical analysis and its interpretation appropriate?

Yes

Are all the source data underlying the results available to ensure full reproducibility?

Yes

Is the study design appropriate and is the work technically sound?

Yes

Are the conclusions drawn adequately supported by the results?

Yes

Are sufficient details of methods and analysis provided to allow replication by others?

Yes

Reviewer Expertise:

Health Economics

I confirm that I have read this submission and believe that I have an appropriate level of expertise to confirm that it is of an acceptable scientific standard.

# Response

**Published:** 2023-04-20

**Authors:** Laia Cirera

**Affiliations:** Barcelona Institute for Global Health, Spain

We thank the reviewer for appreciating the study and for the comments. Please, see below our answers to the comments posed:

 I suggest authors to explore more deeply the contexts of these four high-burden countries in SSA to better interpret the results (e.g. seasonality of transmission, major differences across study countries in health system characteristics, contextual factors, co-payment mechanisms between patients and providers, etc.). The Discussion needs to further explain these differences, which represent a rare opportunity to cost malaria care in pregnancy in low-income countries that have not received much attention in the literature. Despite these differences, is there a key message to policy makers emanating from these four settings?

- **Response: **A full description of the setting has been provided in a separate manuscript (Gonzalez et al - Reference 11). We agree with the reviewer that the intervention areas included in this study are representative of the geographical diversity across the African region and reflect some of the hardest places to reach (in poor and remote contexts). Despite the differences across study areas, our results do provide a clear message to policymakers and emphasize the importance of investing in strategies that reduce the economic burden faced by pregnant women and improve access to malaria care in pregnancy. To emphasize this message, at the beginning of the conclusion the following sentence has been added (line 330): ”Despite the contextual differences across study areas, results from this study demonstrate the significant economic burden that malaria infection imposes on both the household and the health system in endemic countries of SSA.”  

The exit survey questionnaire to pregnant women has been administered when leaving a routine ANC visit from October 2020 to June 2021. The COVID-19 pandemic could have major adverse effects on the provision of health services for other major infectious diseases, such as malaria. Any assumptions about how the programmes for malaria prevention and treatment have been affected during this period of time? Care seeking reduced compared with pre-pandemic levels, prevention services partially suspended (LLIN mass campaigns halted, mass drug distribution or seasonal malaria chemoprevention interrupted, etc.)?

- **Response: **We see the reviewer’s point. The COVID-19 outbreak in Mozambique occurred during the study data collection (October 2020 to June 2021) and therefore, these results have been seen in light of such a big shock, which might have affected our results. Despite this, the implementation of the study went ahead (C-IPTp distribution) and the intervention (adopting full safety measures) continued to be implemented without major disruptions.

Please provide the exclusion criteria of participants: was there an age, co-morbidity limit?

- **Response:** We agree with the reviewer. The inclusion criteria of participants has been clarified and the 2 ^nd^ paragraph of the “Study design and participants” now reads as (lines 71-72): “As such, only women of reproductive age, being pregnant and after leaving a routine ANC visit were selected to participate.    

The costs data have been obtained from a cross-sectional survey, a limitation of the study that has been underlined by the authors. Please explain briefly what data ideally would allow scholars to estimate the economic burden of malaria over the course of the entire malaria season and/or individual pregnancies to better reflect the impact of multiple episodes?

- **Response:** A longitudinal study following up a cohort of pregnant women over the malaria season would have been ideal to effectively capture the full costs of malaria in pregnancy and reflect the economic burden of multiple infections during the same pregnancy. To emphasize this, in the discussion the following sentence has been added (lines 325-326): “A longitudinal study design would have better captured the full costs of malaria in pregnancy.”
